# SIRT1 regulates hepatocyte programmed cell death via GSDME - IL18 axis in human and mouse liver transplantation

**DOI:** 10.1038/s41419-023-06221-0

**Published:** 2023-11-23

**Authors:** Kentaro Kadono, Hidenobu Kojima, Siyuan Yao, Shoichi Kageyama, Kojiro Nakamura, Hirofumi Hirao, Takahiro Ito, Kenneth J. Dery, Douglas G. Farmer, Fady M. Kaldas, Xiaoling Li, Jerzy W. Kupiec-Weglinski

**Affiliations:** 1 Dumont-UCLA Transplantation Center, Department of Surgery, Division of Liver and Pancreas Transplantation, David Geffen School of Medicine at UCLA, Los Angeles, CA 90095 USA; 2 Division of Hepato-Biliary-Pancreatic Surgery and Transplantation, Department of Surgery, Graduate School of Medicine, Kyoto University, Kyoto, Japan; 3 Signal Transduction Laboratory, National Institute of Environmental Health Sciences (NIEHS), Research Triangle Park, NC 27709 USA

**Keywords:** Immune cell death, Prognostic markers

## Abstract

Sirtuin 1 (SIRT1) is a histone/protein deacetylase in the cellular response to inflammatory, metabolic, and oxidative stressors. We previously reported that myeloid SIRT1 regulates the inflamed liver’s canonical pyroptosis cell death pathway. However, whether/how hepatocyte SIRT1 is engaged in programmed cell death in the cold-stressed liver remains uncertain. Here, we undertook translational studies in human and mouse orthotopic liver transplantation (OLT) to interrogate the significance of hepatocyte-specific SIRT1 in cold-stored donor livers and liver grafts after reperfusion. In the clinical arm of sixty human OLT patients, hepatic SIRT1 levels in cold-preserved donor livers correlated with the anti-apoptotic Bcl-2 expression. After reperfusion, improved OLT function was accompanied by hepatic SIRT1 levels negatively associated with cleaved caspase-3 expression. In the experimental arm, we compared FLOX-control with hepatocyte-specific SIRT1-KO livers after orthotopic transplantation into WT mouse recipients, parallel with primary murine hepatocyte cultures subjected to cold activation with/without knockdown of SIRT1, GSDME, and IL18Rβ. Indeed, hepatocyte SIRT1 deficiency upregulated apoptosis and GSDME-mediated programmed cell death, deteriorating hepatocellular function and shortening OLT survival. Augmented GSDME processing, accompanied by increased secretion of IL18 by stressed hepatocytes, was prominent in SIRT1-deficient, cold-stored livers. Hepatocyte SIRT1 expression regulated anti-apoptotic Bcl-2/XIAP proteins, suppressed cold stress-triggered apoptosis, and mitigated GSDME licensing to release IL18. Notably, consistent with the ability of IL18 to depress hepatocyte SIRT1 and Bcl-2/XIAP in vitro, IL18 neutralization in vivo prevented hepatocellular damage and restored the anti-apoptotic phenotype in otherwise injury-prone SIRT1-deficient OLTs. In conclusion, this translational study identifies a novel hepatocyte SIRT1-IL18 molecular circuit as a therapeutic target in the mechanism underpinning hepatocyte death pathways in human and mouse liver transplantation.

## Introduction

Orthotopic liver transplantation (OLT) is a therapy of choice for end-stage liver diseases and certain hepatic malignancies [1]. Despite improvements in patient survival (about 75% at 5-yrs) as a result of advances in immunosuppression and medical management, liver ischemia-reperfusion injury (IRI), an innate immune-driven peritransplant response, represents a major risk factor for clinical outcomes and contributes to the shortage of organs available for life-saving transplantation. Notably, no therapeutic strategy has emerged to prevent IRI in transplant patients. Although the consequences of hepatic IRI in OLT, and potentially in other disease states involving ischemic injury/sterile inflammation, such as stroke and myocardial infarction, are vast, the mechanistic underpinnings leading to IRI are poorly understood.

The liver transplantation procedure encompasses two separate but interrelated stages of ex vivo cold preservation after organ procurement and reperfusion after completing surgery in the recipient. The latter has been extensively studied in mice, but the widely used local warm liver IRI model lacks the cold-ischemia component [2]. This is a flaw because cold organ storage triggers hepatocellular damage independently [3]. Liver sinusoidal endothelial cells (LSEC) are particularly susceptible to cold stress [4], and maintaining hepatocyte integrity is essential for IRI resistance [5]. Indeed, hepatocyte rather than LSEC injury markers in the liver wash-out may better predict early graft viability [6]. While we have shown that the stress-responsive ASK1-p38 axis is involved in cold storage-triggered OLT damage [7], a complete roadmap of molecular events, particularly cell death pathways, initiated by the cold stress is needed.

Consistent with the cell death program being an essential determinant in OLT outcomes, we have reported that the Bcl-2/Bcl-xL anti-apoptotic phenotype is critical for IRI resistance in murine OLT [8]. However, the specific cell death cascades that operate during liver IRI remain uncertain [9]. Historically, apoptosis has been considered a non-inflammatory cell death program, while necrosis is a cytokine-mediated inflammatory process, and each was thought to proceed independently in a mutually exclusive manner. Recent studies, however, challenge this concept because apoptosis can crosslink with necroptosis and pyroptosis [10]. Indeed, caspase-3, a key apoptosis executor, may activate Gasdermin E (GSDME), leading to a secondary programmed cell death (PCD), accompanied by the release of danger-associated molecular patterns (DAMPs), such as high-mobility group box 1 (HMGB1) and inflammatory cytokines [11, 12]. Moreover, caspase-8, which mediates extrinsic apoptosis, may also control a switch between apoptosis, necroptosis, and pyroptosis [13]. How these pathways communicate in IR-triggered sterile inflammation in OLT recipients remains unknown.

To unravel molecular events initiated by liver cold preservation, we have focused on Sirtuin1 (SIRT1; silent mating type information regulation 2 homolog 1), one of the class III histone deacetylases involved in the cellular senescence, inflammation, and stress resistance. Recently, we discovered the protective function of myeloid SIRT1 [14, 15] and its negative regulation of canonical inflammasome-pyroptosis in human and murine OLT [16], while others have shown that hepatocyte SIRT1 deficiency impaired lipid homeostasis and promoted steatosis in mouse livers under high-fat diet [17]. As the cleavage of hepatocyte Gasdermin D (GSDMD) by caspase-1, which defines the canonical pyroptosis pathway, failed to affect liver IRI [18], while myeloid SIRT1 attenuated liver IRI by suppressing caspase-1 – GSDMD activation axis [16], we asked as to whether and how hepatocyte SIRT1 might regulate PCD in IR-stressed OLT.

## Results

### Human hepatic SIRT1 expression correlates with the apoptotic phenotype in clinical OLT

**Fig. 1:**
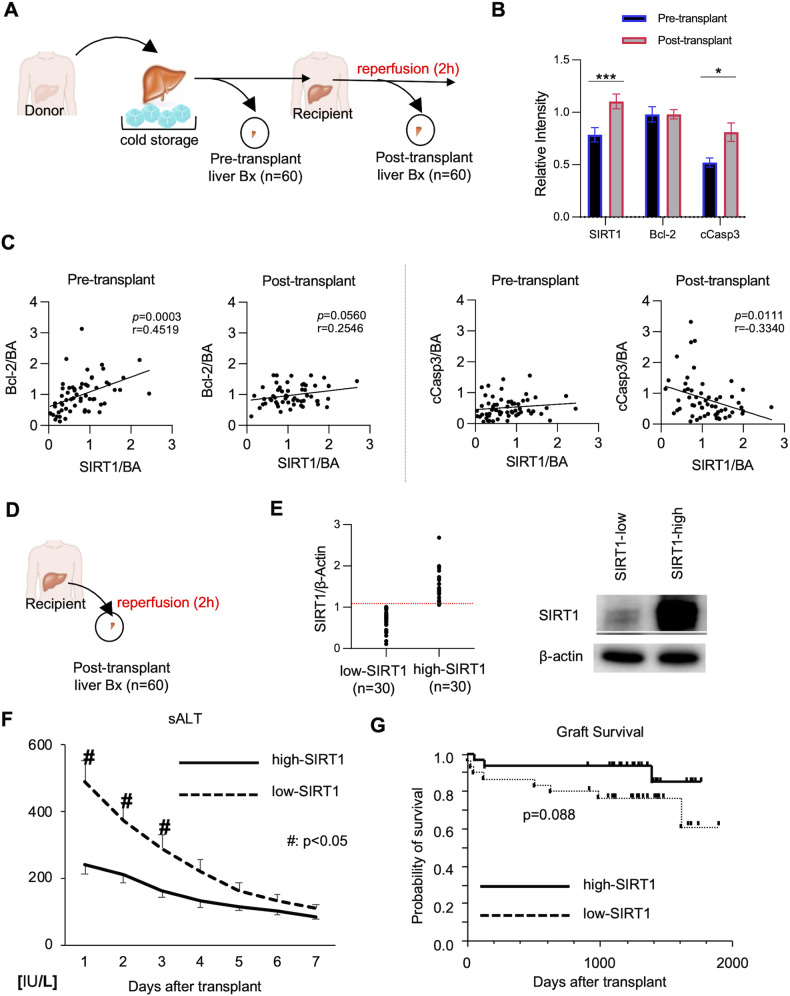
Hepatocyte SIRT1 regulates apoptosis and hepatocellular function in clinical OLT. **A** Human liver biopsies (Bx; *n* = 60) were collected at the back-table before transplantation (after cold storage) and after liver transplantation at 2 h post-reperfusion (before abdominal closure). **B** WB-assisted detection of hepatic SIRT1, Bcl-2, and cCasp3 with β-actin normalization. Data are shown in bars indicative of mean ± SEM. Statistical analyses with 2-tailed Man–Whitney *U* test. *p < 0.05; **p < 0.01. **C** The correlation between SIRT1 and Bcl-2/cCasp3 levels in pre-transplant (*left panel*) and post-transplant (*right panel*) liver Bx samples was analyzed by nonparametric Spearman’s method. r, Spearman’s correlation coefficient. **D** Human OLT Bx samples collected at 2 h after reperfusion were divided into (**E**) low (*n* = 30) and high (*n* = 30) SIRT1 expression groups, based on the relative WB-assisted expression of SIRT1/β-actin. **F** Serum ALT levels at POD 1-7 in OLT recipients. ^#^*P* < 0.05 (Mann–Whitney *U* test) (**G**) The cumulative probability of overall OLT survival based on hepatic SIRT1 levels (Kaplan–Meier method). Solid line: high-SIRT1; dotted line: low-SIRT1 group (log-rank test).

We evaluated retrospectively perioperative hepatic SIRT1 expression and its correlation with hepatocellular function in a clinical cohort of sixty OLT patients. Human liver biopsies (Bx) were collected after cold storage at the back table (before transplant surgery), and post-transplant liver Bx were obtained at about two hours post-reperfusion (before abdomen closure) (Fig. 1A). Although SIRT1 regulates apoptosis cell death [19], its relationship with the apoptotic program in human OLT remains elusive. Unlike enhanced levels of cCasp3 (*P* < 0.05), anti-apoptotic Bcl-2 remained unchanged in human OLT (Fig. 1B). Indeed, SIRT1 levels correlated positively with Bcl-2 (r = 0.4519; *P* = 0.0003) in pre-transplant, and negatively with cCasp3 (r = −0.3340; *P* = 0.0111) in post-reperfusion Bx, indicating SIRT1 expression is associated with apoptosis in human OLT.

To evaluate the impact of graft SIRT1 on clinical outcomes, we classified sixty human OLTs into SIRT1**-**low and SIRT1-high expression groups (Fig. 1E). Patients’ demographic data and clinical parameters are shown (Supplementary Table 1/2). There was no correlation between SIRT1 levels and recipient/surgical parameters, including age, gender, race, BMI, disease etiology, ABO compatibility, MELD score, pre-transplant blood tests, pre-operative hospital stay, cold/warm ischemia time, or blood transfusions during the surgery (Supplementary Table 1). There was no correlation between SIRT1 grouping and donor data (Supplementary Table 2), including age, gender, race, BMI, pre-procurement blood tests, and donation status (after circulatory or brain death).

Compared to the SIRT1-low expression group, SIRT1-high OLT patients showed improved hepatocellular function, evidenced by decreased sALT levels at postoperative days 1–3 (*P* < 0.05; Fig. 1F). We next analyzed cumulative OLT survival, with median follow-up at 1280 days (range, 3-1892 days). Compared to the SIRT1-low clinical cohort, the SIRT1-high expression group displayed a noticeable trend of improved OLT survival even though it failed to reach statistical significance (Fig. 1G). This data suggests that hepatic SIRT1 expression may improve early OLT function and affect clinical outcomes.

### Hepatocyte SIRT1 improves mouse OLT and suppresses apoptosis with GSDME licensing

**Fig. 2:**
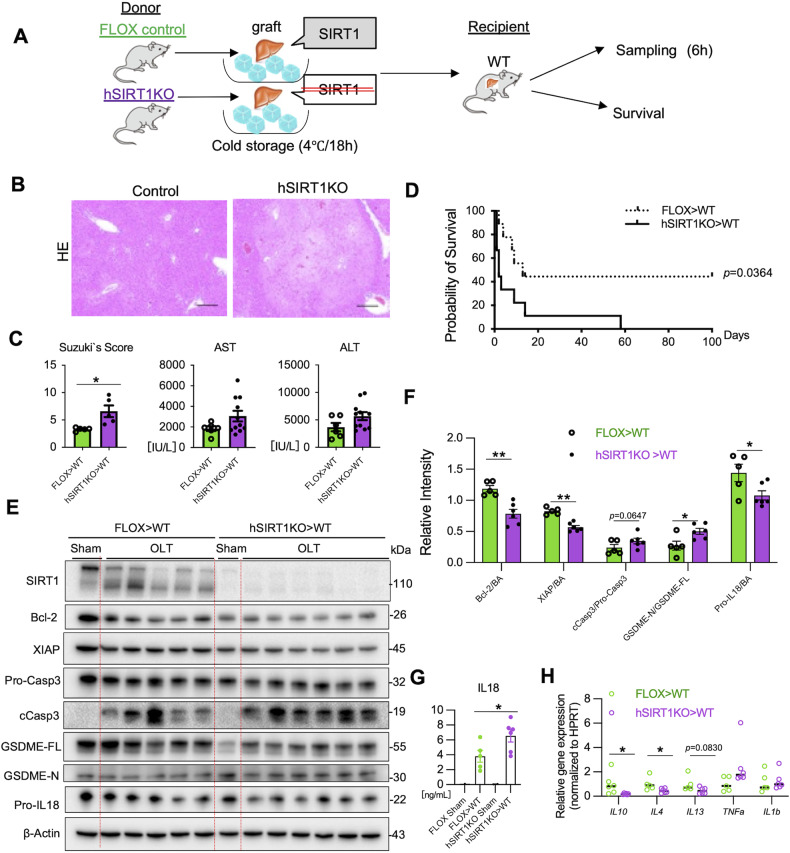
Hepatocyte SIRT1 deficiency exacerbates apoptosis and GSDME processing in mouse OLT. **A** Livers from FLOX control and hSIRT1KO mice were stored in UW solution (18 h/4 °C) and transplanted into WT mice, followed by OLT sampling at 6 h post-reperfusion. A separate group of OLT recipients was monitored for survival. **B** Representative OLT staining (H&E; original magnification ×100; scale bar: 200 μm). **C** sAST, sALT levels, and Suzuki’s histological score of liver IRI. **D** The cumulative OLT survival (Kaplan-Meier method). Dotted line: FLOX > WT; solid line: hSIRT1KO>WT (*n* = 9/group). **E** WB-assisted detection of SIRT1, Bcl-2, XIAP, Pro-Casp3, cCasp3, GSDME-FL, GSDME-N, Pro-IL18, and β-Actin in OLT. **F** The relative intensity ratios of Bcl-2, XIAP, cCasp3/Pro-Casp-3, GSDME-N/GSDME-FL, and Pro-IL18 normalized with β-Actin in OLT. **G** ELISA-assisted detection of serum IL18 levels. **H** qRT-PCR-assisted detection of mRNA coding for IL10, IL4, IL13, TNFα, and IL1β in OLT. Data were normalized to HPRT (*n* = 5-6/group). Data shown are mean ± SEM. *p < 0.05; **p < 0.01 by Student’s *t*-test.

To gain a better understanding of how hepatocyte SIRT1 may affect clinical outcomes, we applied a well-established murine model of extended ex vivo cold liver preservation (18 h) followed by OLT, which mimics marginal human transplant setting (Fig. 2A). At 6 h post-reperfusion, the peak of hepatocellular injury in this model, hSIRT1-deficient livers transplanted into WT recipients showed exacerbated sinusoidal congestion, vacuolization, and hepatocellular necrosis compared with controls (Fig. 2B, C; Suzuki’s score: hSIRT1KO>WT = 6.6 ± 1.06 vs. FLOX > WT = 3.31 ± 0.14, *P* < 0.05). These findings correlated with deteriorated hepatocellular function (sAST [IU/L]: hSIRT1KO>WT = 3062 ± 521 vs. FLOX > WT = 1843 ± 234, *P* = 0.1182 and sALT [IU/L]: hSIRT1KO>WT = 5690 ± 730 vs. FLOX > WT = 3689 ± 767, *P* = 0.1008) (Fig. 2C), and worsened OLT survival (day 100: hSIRT1KO>WT = 0% vs. FLOX > WT = 40%), *P* < 0.05 (Fig. 2D).

Having shown the association between SIRT1 expression and apoptotic cell death in OLT patients (Fig. 1), we next evaluated the apoptotic-pyroptotic activation profile in a murine OLT model. Indeed, hepatocyte SIRT1 deficiency decreased levels of Bcl-2 (*P* < 0.01) and X-chromosome linked inhibitor of apoptosis protein (XIAP), the caspase inhibitor [20] (*P* < 0.01) (Fig. 2E, F). Moreover, hepatic SIRT1 deficiency increased levels of cCasp3, and the N-terminal GSDME fragment (GSDME-N) (*P* < 0.05) while decreasing Pro-IL18 (*P* < 0.05) in liver cell lysates (Fig. 2E, F). In parallel, increased levels of mature bioactive IL18 were detected in sera samples by ELISA (FLOX > WT = 3.806 ± 0.8037 vs. hSIRT1KO>WT = 6.540 ± 0.8278 ng/mL, *P* < 0.05; Fig. 2G). In the qPCR-assisted screening, disruption of hepatocyte SIRT1 suppressed IL10, IL4, and IL13 while increasing TNFα levels in OLTs (Fig. 2H). These data are consistent with the idea that hepatocyte SIRT1 regulates IR-triggered liver inflammation and attenuates OLT damage by suppressing caspase-3 – GSDME-dependent PCD.

### Cold storage triggers GSDME-mediated PCD in mouse livers

**Fig. 3:**
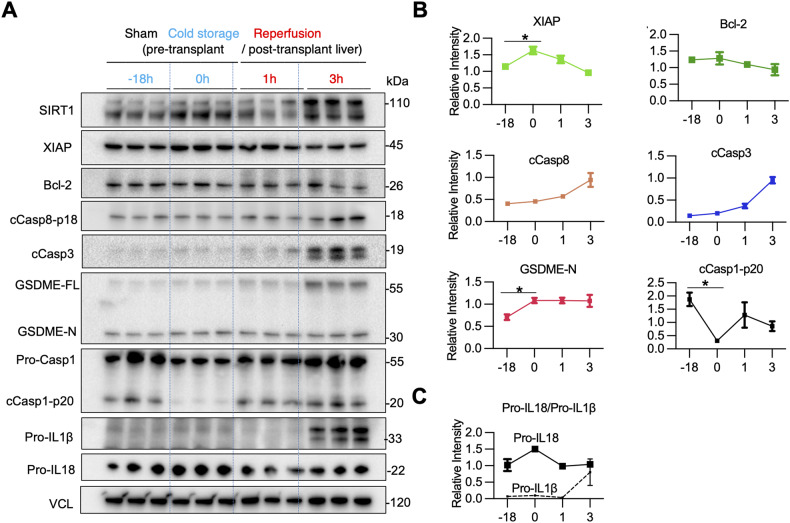
Cold preservation of mouse livers triggers GSDME processing in stressed hepatocytes. WT livers stored in UW solution (18 h/4 °C) were transplanted into syngeneic hosts. Hepatic samples were collected after cold storage (pre-transplant; 0 h) and post-transplantation (1 h, 3 h). **A** WB-assisted detection of hepatic SIRT1, XIAP, Bcl-2, cCasp8, cCasp3, GSDME-FL, GSDME-N, Pro-Casp-1, cCasp1-p20, Pro-IL1β, Pro-IL18, and VCL. **B** Kinetics of relative intensity for hepatic XIAP, Bcl-2, cCasp8, cCasp3, GSDME-N, and cCasp1-p20 normalized with VCL. Data shown are mean ± SEM. *p < 0.05; **p < 0.01 by Student’s *t*-test. **C** The relative intensity in Pro-IL18 (solid line), and Pro-IL1β (dotted line) in mouse livers.

To determine the kinetics of apoptosis-GSDME processing, we compared cold-preserved WT livers (4 °C/18 h) with those after transplantation (1 h and 3 h post-OLT) (Fig. 3A, B). The pyroptosis executor, GSDME-N, increased after cold preservation compared to sham controls (*P* < 0.05). Although cCasp3 is a major GSDME activator, increased levels of pro-apoptotic cleaved caspase-8 (cCasp8) (*P* < 0.01), and cCasp3 were observed in transplanted livers at reperfusion, while anti-apoptotic XIAP protein significantly increased already after cold storage. In contrast, cold stress alone markedly reduced cleaved caspase-1 (cCasp1), a marker of the canonical inflammasome-pyroptosis (Fig. 3A, B). Unlike Pro-IL1β, which increased remarkably at 3 h post-OLT, Pro-IL18 was expressed throughout cold storage and reperfusion periods (Fig. 3A, C), consistent with IL18, but not IL1β, being constitutively expressed at the transcriptional and protein levels [21]. Hence, IL18 becomes operational in IRI-OLT at a much earlier phase than IL1β, while GSDME-processing is already activated in cold-stressed livers before transplantation.

### Hepatocyte SIRT1 suppresses apoptosis and GSDME-mediated PCD in cold-stored mouse livers

**Fig. 4:**
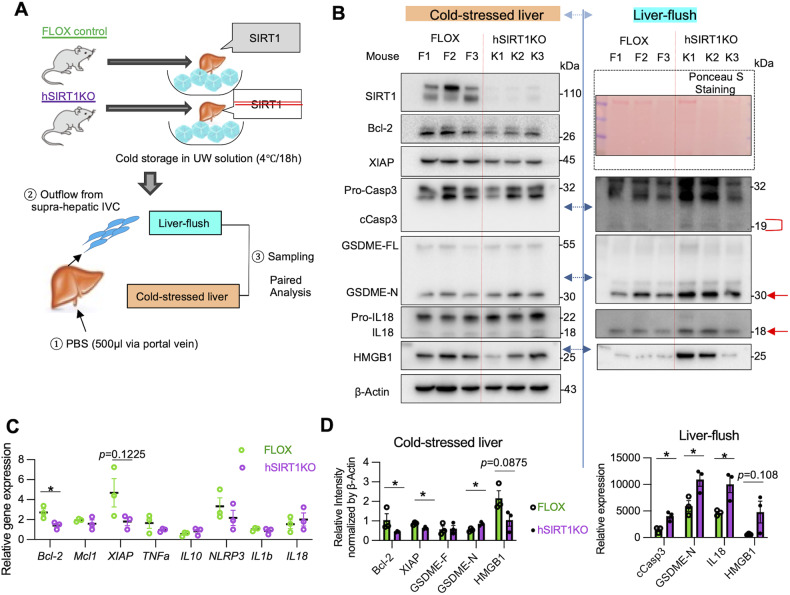
Hepatocyte SIRT1 suppresses apoptosis and GSDME-mediated PCD in cold-stored mouse livers. **A** Groups of FLOX and hSIRT1KO livers cold-stored in UW solution were perfused with physiological saline (0.5 ml) via a portal vein-cuff to collect liver flush from supra-hepatic inferior vena cava (*n* = 3/group). **B** WB-assisted detection of SIRT1, Bcl-2, XIAP, Pro-Casp3, cCasp3, GSDME-FL, GSDME-N, Pro-IL18, IL18, and β-actin in cold-stored livers (*left panel*). Some targets were evaluated in the liver flush by WB (*right panel*). **C** qRT-PCR assisted detection of mRNA coding for Bcl-2, Mcl1, XIAP, TNFα, IL10, NLRP3, IL1β, and IL18. **D** The relative intensity of Bcl-2, XIAP, GSDME-F, and GSDME-N normalized with β-actin in cold-stressed liver (*left panel*). The relative intensity of IL18, cCasp3, and GSDME-N in the liver flush (*right panel*). Data shown are mean ± SEM. *p < 0.05 by Student’s t-test.

We next asked whether hepatocyte SIRT1 regulates apoptosis and GSDME-mediated PCD during cold liver storage. By reflecting the degree of tissue damage, liver enzymes released into the washout serve as a surrogate measure of post-reperfusion graft function [22]. In addition, specific activated cell death executor proteins released into the extracellular space may be detected in the hepatic washout. Thus, we analyzed liver flush in parallel with hepatic tissue in our ex vivo preservation setting (Fig. 4A). Consistent with pre-transplant human Bx samples (Fig. 1C), hSIRT1-deficient cold-stressed mouse livers showed decreased gene/protein expression of Bcl-2 and XIAP (Fig. 4B, C, D) but unchanged cCasp3 levels, compared with FLOX controls (Fig. 4B). At the same time, GSDME-N trended higher in hSIRT1-KO livers, and the liver flush (*P* < 0.05) (Fig. 4B), suggesting GSDME-N may mediate PCD in cold-stored SIRT1-deficient livers. Concomitant up-regulation of cCasp3 in the liver flush confirms GSDME cleavage by cCasp3, one of its two principal proteases [11]. Interestingly, we found higher levels of IL18, one of the standard mediators of GSDME activation [23], in the liver flush from SIRT1-deficient compared to FLOX livers, with little or no mature bioactive IL1β (Supplementary Fig. 1). There were no changes in the canonical inflammasome activation profile between FLOX and hSIRT1-deficient cold-stressed livers, evidenced by comparable levels of cleaved caspase-1-p20 (cCasp1-p20) and GSDMD-N. The ability of hepatocyte SIRT1 to promote Bcl-2/XIAP and prevent GSDME from releasing IL18 in mouse livers was confirmed in discarded cold-stored human livers (Supplementary Fig. 2).

### Hepatocyte SIRT1 deficiency promotes apoptosis and GSDME in vitro

**Fig. 5:**
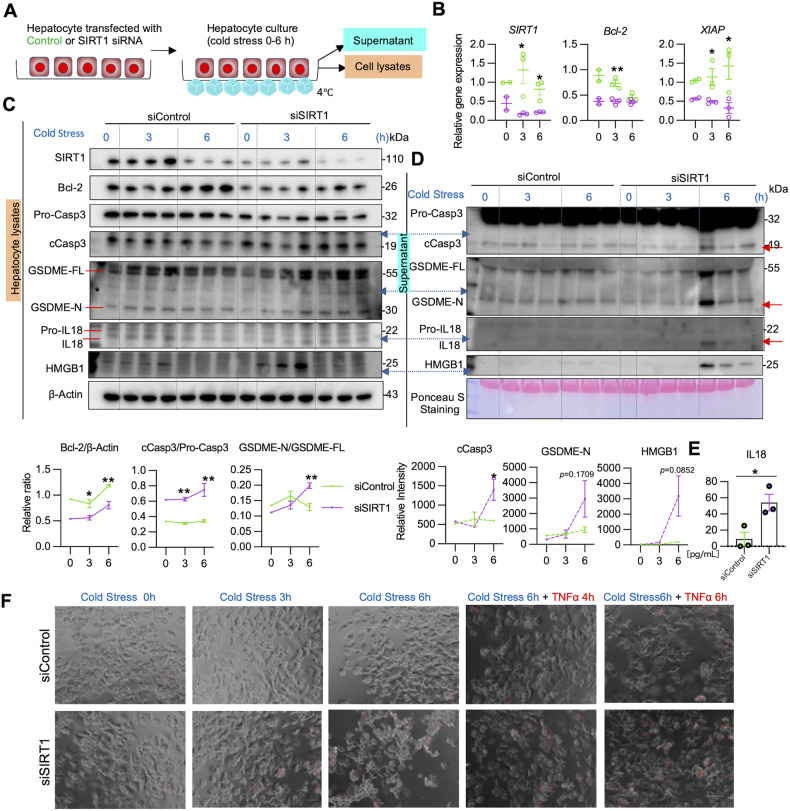
Hepatocyte SIRT1 deficiency depresses the anti-apoptotic gene program and promotes GSDME processing under cold stress in vitro. **A** Primary mouse hepatocytes transfected with SIRT1 siRNA vs. control siRNA were subjected to cold stimulation (0–6 h). **B** qRT-PCR assisted detection of mRNA coding for SIRT1, Bcl-2, and XIAP. Green dots: siControl; purple dots; siSIRT1. **C** WB-assisted detection of SIRT1, Bcl-2, Pro-Casp3, cCasp3, GSDME-FL, GSDME-N, Pro-IL18, IL18, and β-actin in hepatocyte lysates (*upper panels*). The relative intensity ratios of Bcl-2, cCasp3/Pro-Casp3, and GSDME-N/GSDME-FL (*lower panels*). Green line: siControl; purple line: siSIRT1. **D** WB-assisted detection of Pro-Casp3, cCasp3. GSDME-FL, GSDME-N, Pro-IL18, and IL18 in the culture medium (*upper panels*). Ponceau S staining is shown as a loading control. The relative intensity ratios of cCasp3 and GSDME-N (*lower panels*). Green line: siControl; purple line: siSIRT1. **E** ELISA-assisted IL18 levels in cold-stressed hepatocytes. Data shown are mean ± SEM. *p < 0.05; **p < 0.01 by Student’s *t*-test. **F** Composite images of phase contrast and immunofluorescence propidium iodide staining of primary mouse hepatocytes transfected with control (*upper panels*) or SIRT1 (*lower panels*) siRNAs under cold stress (3 h, 6 h), followed by TNFα stimulation (4 h or 6 h; 25 ng/mL). Original magnification ×200; scale bar; 100 μM.

To further elucidate how SIRT1 regulates hepatocyte apoptosis and GSDME-mediated PCD, we screened for the expression of apoptotic and pyroptotic markers in primary mouse hepatocyte cultures, with/without siRNA-silencing of SIRT1 upon cold stimulation (Fig. 5A). SIRT1-deficient hepatocytes showed suppressed Bcl-2/XIAP gene expression in vivo (Fig. 5B), while SIRT1-silencing down-regulated hepatocyte Bcl-2 protein but upregulated cCasp3/GSDME levels in vitro (Fig. 5C). Consistent with cold-stored livers (Fig. 4), SIRT1-silenced hepatocyte cultures secreted significant amounts of IL18 (*P* < 0.05; Fig. 5D, E), concomitantly with cleaved forms of cCasp3/GSDME (Fig. 5D). These data imply that SIRT1 promotes anti-apoptotic Bcl-2 and XIAP proteins to prevent cold stress-induced cell death cascade. Notably, although hepatocytes have never been reported to secrete IL18, we observed cold-stressed SIRT1-deficient hepatocytes to release ample IL18 (Fig. 5D, E).

We also evaluated the expression of p53 transcription factor, an essential SIRT1 target regulated by acetylation/deacetylation (14). Indeed, SIRT1 ablation upregulated acetylation and phosphorylation of p53 protein in cold-stressed primary mouse hepatocyte cultures (Supplementary Fig. 3), confirming that SIRT1 can regulate p53 via a deacytelase function.

Whether hepatocytes can undergo pyroptosis regardless of gasdermin activation remains controversial [24]. Although in our study, SIRT1-silenced hepatocytes displayed up-regulated cell membrane permeability, evidenced by increased propidium iodide (PI) uptake, we observed cell shrinkage rather than swelling or rupture, cardinal morphologic features of hepatocyte pyroptosis (Fig. 5F). To mimic the OLT reperfusion phase, we then supplemented cold-stressed hepatocyte cultures with a TNFα adjunct. Indeed, unlike in controls, the addition of TNFα readily triggered PCD in SIRT1-silenced hepatocytes (Fig. 5F).

### SIRT1 regulates cold stress-induced apoptosis and GSDME licensing to release IL18

**Fig. 6:**
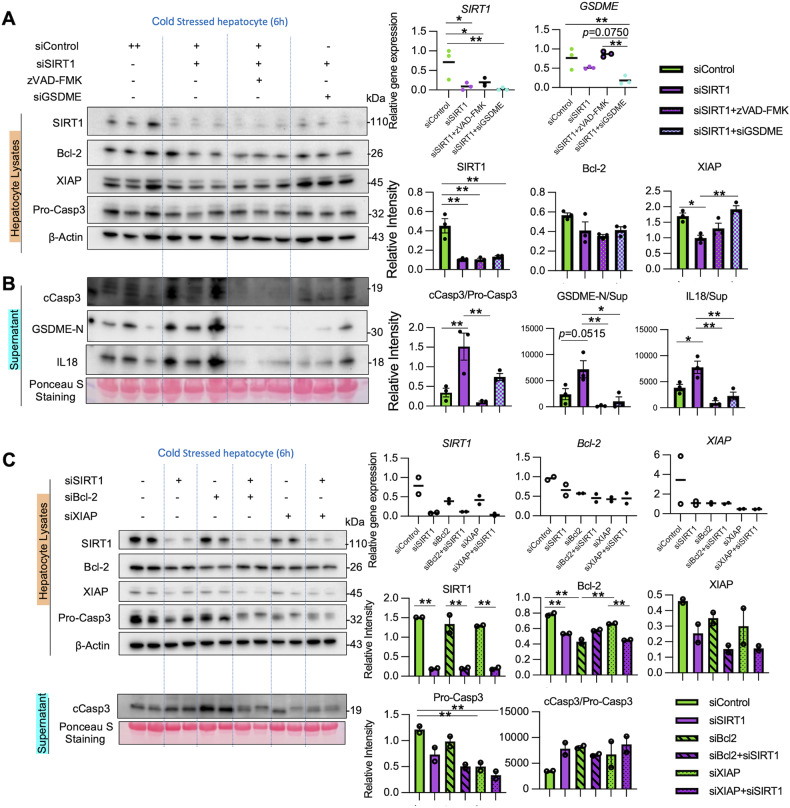
SIRT1 regulates cold stress-induced apoptosis and GSDME processing to release IL18 in vitro. **A**, **B** Primary mouse hepatocytes transfected with control or SIRT1 siRNA were subjected to cold stress (6 h). Some SIRT1-silenced cells were pretreated with a pan-caspase inhibitor zVAD-FMK (20 nM, 18 h) or transfected with GSDME siRNA. **A** WB-assisted detection of SIRT1, Bcl-2, XIAP, Pro-Casp3, and β-actin in lysates (*left panels*). qRT-PCR-assisted detection of mRNA coding for SIRT1 and GSDME (*right upper panels*). The relative intensity of SIRT1, Bcl-2, and XIAP in lysates (*right lower panels*). **B** cCasp3, GSDME-N, and IL18 in supernatants (*left panels*). The relative intensity of cCasp3/Pro-Casp3 ratio, GSDME-N, and IL18 in supernatants (*right panels*). **C** Primary mouse hepatocytes transfected with SIRT1 siRNA or/and Bcl-2 siRNA or/and XIAP siRNA were subjected to cold stress (6 h). WB-assisted detection of SIRT1, Bcl-2, XIAP, Pro-Casp3, and β-Actin in hepatocyte lysates (*left upper panels*) and cCasp3 in supernatants (*left lower panels*). The relative intensity of SIRT1, Bcl-2, XIAP, and cCasp3/Pro-Casp3 ratio (*right panels*). Data shown are mean ± SEM. *p < 0.05; **p < 0.01 by one-way ANOVA.

We used a siRNA approach to address the role of GSDME in hepatocyte PCD under cold stress. Indeed, GSDME-silenced hepatocyte cultures showed remarkable suppression of IL18 and HMGB1 secretion (Supplementary Fig. 4). Furthermore, to elucidate the relationship between SIRT1, apoptosis/GSDME-mediated PCD, and IL18 release, we treated SIRT1-silenced hepatocytes with zVAD-FMK, a pan-caspase inhibitor, or transfected SIRT1-silenced hepatocytes with GSDME-siRNA, before cold stimulation (Fig. 6A, B). First, adjunctive conditioning with zVAD-FMK significantly suppressed hepatocyte GSDME activation, compared to SIRT1-silenced cells alone, indicating GSDME activation under cold stress was caspase-dependent. Second, IL18 release in SIRT1-silenced hepatocytes was reduced after treatment with zVAD-FMK or transfection with GSDME-siRNA. These data suggest that hepatocyte SIRT1 regulates IL18 release in a caspase- and GSDME-dependent manner. Notably, GSDME-silenced hepatocytes displayed increased levels of XIAP compared with SIRT1 silencing alone. This suggests that anti-apoptotic proteins control cell death and PCD may affect the anti-apoptotic protein program.

Next, we investigated whether Bcl-2/XIAP are essential for SIRT1 regulation of caspase activation. With silencing Bcl-2 or XIAP, SIRT1-knock downed hepatocytes failed to augment caspase 3 activation compared to controls (Fig. 6C). Thus, Bcl-2/XIAP are critical for SIRT1 regulation of apoptosis in cold-stressed hepatocytes.

### IL18 suppresses hepatocyte SIRT1 and the anti-apoptotic protein axis

**Fig. 7:**
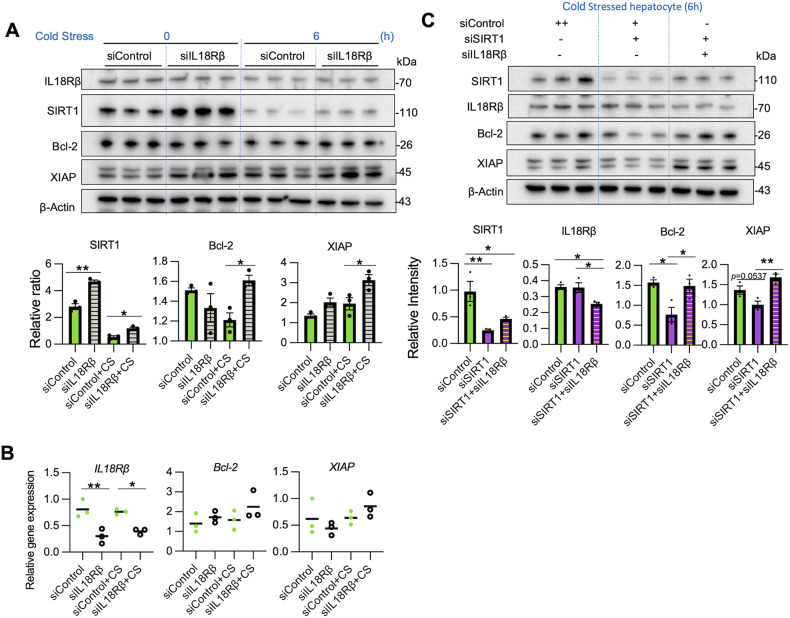
IL18 suppresses hepatocyte SIRT1 and anti-apoptotic axis. Primary hepatocytes transfected with control or IL18Rβ siRNA were subjected to cold stress (0–6 h). **A** WB-assisted detection of IL18Rβ, SIRT1, Bcl-2, XIAP, and β-actin (*upper panels*). The relative ratio of SIRT1, Bcl-2, and XIAP (*lower panels*). **B** qRT-PCR-assisted detection of mRNA coding for IL18Rβ, Bcl-2, and XIAP. **C** Primary hepatocytes transfected with control siRNA or SIRT1 siRNA and/or IL18Rβ siRNA were subjected to cold stress (6 h). WB-assisted detection of SIRT1, IL18Rβ, Bcl-2, XIAP, and β-Actin (*upper panels*). The relative intensity of SIRT1, IL18Rβ, Bcl-2, and XIAP (*lower panels*). Data shown are mean ± SEM. *p < 0.05; **p < 0.01 by one-way ANOVA.

To investigate the effect of IL18 on hepatocytes, we silenced the IL18 receptor β unit (IL18Rβ), which is specific for hepatocyte IL18 response and indispensable for high-affinity IL18 binding [25]. Interestingly, IL18Rβ knockdown up-regulated SIRT1, Bcl-2, and XIAP under cold stress and without IL18 stimulation (*P* < 0.05) (Fig. 7A, B), suggesting that extracellular IL18 can accelerate hepatocyte death. Then, to evaluate whether IL18R regulation of anti-apoptotic factors is SIRT1-dependent, we knock-downed IL18Rβ in SIRT1-silenced hepatocytes. While SIRT1-silencing alone depressed hepatic Bcl-2/XIAP levels, concomitant transfection with IL18Rβ siRNA rescued Bcl-2/XIAP in SIRT1-silenced hepatocytes (Fig. 7C), suggesting IL18R-mediated Bcl-2/XIAP regulation is SIRT1-independent. Thus, IL18 is required for a positive feedback loop where SIRT1 regulates the anti-apoptotic programs in cold-stressed hepatocytes.

### IL18 neutralization mitigates liver damage and restores anti-apoptotic phenotype in OLT

**Fig. 8:**
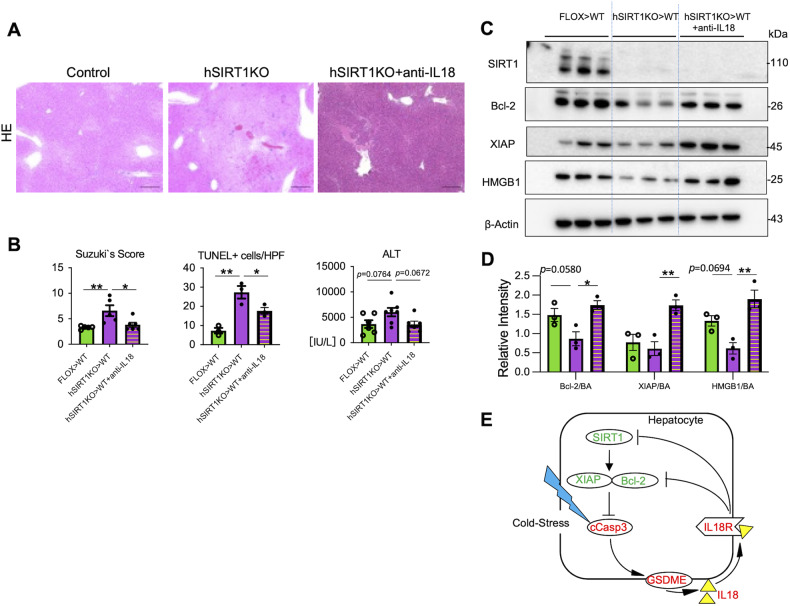
IL18 neutralization prevents liver IRI and promotes anti-apoptotic phenotype in SIRT1-deficient murine OLT. Groups of FLOX control and hSIRT1KO livers, stored in UW solution (18 h/4 °C), were transplanted into WT mice that remained untreated or pretreated with anti-IL18 Ab (0.5 mg i.p. at -1 day), followed by sampling at 6 h post-OLT. **A** Representative H&E staining (original magnification ×100; scale bar 200 μm); **B** Suzuki’s histological score of liver IRI; frequency of TUNEL+ cells/HPF (original magnification ×200; scale bar 100 μm); and sALT levels. **C** WB-assisted detection of SIRT1, XIAP, Bcl-2, HMGB1, and β-actin in OLTs. **D** The relative intensity ratios of Bcl-2, XIAP, and HMGB1 normalized with β-actin. Data shown are for *n* = 5-7/group (sALT/Suzuki’s score) and *n* = 3/group (TUNEL staining/WB); mean ± SEM. *p < 0.05; **p < 0.01 by one-way ANOVA. **E** Schematic illustration of proposed cold stress-triggered hepatocyte cell death programs. SIRT1 regulates the activation of caspase3, followed by GSDME activation to release IL18 via the Bcl-2/XIAP anti-apoptotic axis. The secreted IL18 further down-regulates SIRT1, Bcl-2, and XIAP.

As the IL18R-IL18 activation axis was critical for homeostatic SIRT1 regulation of anti-apoptotic programs in cold-stressed hepatocytes in vitro, we next aimed to assess the in vivo function of IL18 in IR-stressed OLT. Groups of hSIRT1KO cold-stored livers were transplanted to WT mice with/without adjunctive anti-IL18 mAb conditioning. As shown in Fig. 8A, B, by 6 h post-reperfusion, OLTs in IL18-neutralized recipients showed well-preserved histological detail, with decreased sinusoidal congestion, vacuolization, and hepatocellular necrosis (Suzuki’s score: hSIRT1KO>WT+anti-IL18 = 3.83 ± 0.47 vs. hSIRT1KO>WT = 6.6 ± 1.06, *P* < 0.05), lower frequency of TUNEL+ cells/HPF (hSIRT1KO>WT+anti-IL18 = 17.67 ± 1.76 vs. hSIRT1KO>WT = 27.33 ± 3.28, *P* < 0.05), and improved function (sALT [IU/L]: hSIRT1KO>WT+anti-IL18 = 3616 ± 628 vs. hSIRT1KO>WT = 6043 ± 835, *P* = 0.067). Consistent with siIL18Rβ-silenced SIRT1-deficient hepatocyte cultures (Fig. 7C), IL18 deficiency in vivo restored anti-apoptotic phenotype in hSIRT1KO livers, consistent with increased Bcl-2/XIAP levels, and decreased HMGB1 release, indicating OLTs were less vulnerable to IR stress in IL18-deficient environment (Fig. 8C, D).

## Discussion

This translational study uncovered the regulatory function of hSIRT1 in the mechanism of cold stress-induced hepatocyte death in human and mouse OLT recipients. Clinical screening of sixty human liver transplant patients revealed that increased SIRT1 expression correlated positively with the anti-apoptotic cell death phenotype and negatively with the apoptotic pathway while improving the early hepatocellular function. The experimental arm established a hepatocyte cell death feedback loop in mouse OLT recipients, where GSDME licensed extracellular IL18 to depress the inflamed liver’s homeostatic SIRT1 and anti-apoptotic phenotype.

We have previously shown that by up-regulating SIRT1 expression, treatment of mice with Resveratrol, a natural polyphenolic phytoalexin, decreased proinflammatory cytokine programs, both in vitro and in vivo, as well as attenuated leukocyte infiltration and the hepatocellular damage in a murine model of liver warm IRI (15). We have recently reported that myeloid-specific SIRT1 regulated hepatic inflammation and the canonical inflammasome assembly in IR-stressed livers [16]. Having confirmed the decrease of Pro-IL18 in IR-stressed mouse OLT and increased IL18 levels in serum and the liver flush under cold stimulation (Figs. 2H, 4B), we expected SIRT1 to exert a similar regulatory function in the stressed hepatocytes. However, with no difference in caspase-1/GSDMD expression between FLOX-control and hSIRT1-deficient livers (Supplementary Fig. 1/5), we noticed increased apoptosis and GSDME-mediated PCD selectively in hSIRT1-deficient livers. Notably, unlike hepatocyte SIRT1 deficiency, disruption of myeloid SIRT1 did not affect apoptosis in IR-stressed livers (Supplementary Fig. 6). To the best of our knowledge, this is the first report to document the GSDME-mediated PCD pathway in OLT recipients.

GSDME of the gasdermin family initiates pyroptosis following cleavage by caspase-3 [11], and it is also essential for releasing DAMPs, such as HMGB1 [12, 23], and inflammatory cytokines, such as IL1β, IL18 [26], and IL1α [27]. Here, hSIRT1-deficient cold-stored livers in vivo, and SIRT1-silenced hepatocytes in vitro were enriched in IL18 but not mature bioactive IL1β. Moreover, siRNA-facilitated silencing confirmed that hepatocyte GSDME regulated IL18 release under cold stress conditions. In contrast to IL1β, which requires amplifying the NF-κB precursor for activation, IL18 is constitutively expressed in unstimulated cells [28]. Therefore, detecting hepatocyte-derived IL18 rather than IL1β was unsurprising, although no previous reports described stressed hepatocytes releasing IL18. Indeed, IL18 has been primarily associated with activating NK/Th1 cells and IFN-γ production in response to intracellular infection [29]. Although IL18 induces extrinsic apoptosis in hepatocytes [30] and endothelial cells [31], GSDME-silenced hepatocytes showed increased XIAP with decreased IL18 levels in our study, indicating that GSDME-licensed IL18 depressed anti-apoptotic protein program. Rogers et al. reported that GSDME augmented apoptosis in lymphoid cell lines/macrophages by forming mitochondrial pores independently of Bid truncation, a pro-apoptotic protein of the Bcl-2 family [23]. As GSDME processing down-regulated Bcl-2/XIAP to activate caspase-3, IL18Rβ-silenced hepatocytes had significantly increased anti-apoptotic protein levels (Fig. 7A, B). Mouse hSIRT1-deficient recipients showed higher serum and lower hepatic IL18 levels, with augmented apoptosis and GSDME processing. Consistent with that finding, Bysani et al. demonstrated that endothelial cell-derived IL18 might induce inflammation and apoptosis in myocardial cells via autocrine and paracrine fashion [31].

Hence, we have identified a novel mechanism by which GSDME-licensed IL18 bridges the apoptosis feedback loop in cold-stressed hepatocytes. Although further studies are needed to elucidate how IL18 can augment hepatocyte apoptosis, our data are consistent with the idea that hepatocyte-derived IL18 should be regarded as a new player in the pathogenesis of inflammatory liver diseases. Strikingly, while IL18 readily depressed hepatocyte SIRT1 and Bcl-2/XIAP expression in vitro, neutralization of IL18 in vivo exerted beneficial biological function by mitigating IR-triggered hepatocellular damage and restoring the anti-apoptotic phenotype in otherwise injury-prone SIRT1-deficient OLTs (Fig. 8).

Although cCasp3 activates GSDME-mediated PCD, a recent study showed that granzyme B could process GSDME in cytotoxic CD8 T lymphocytes [32]. While hSIRT1 deficiency in the donor liver (KO) augmented cCasp3, the kinetic analysis showed apoptosis occurred primarily after reperfusion in SIRT1-proficient livers (WT), accompanied by up-regulation of XIAP during cold stress (Fig. 3). In agreement with augmented caspase3/GSDME activation, SIRT1 deficiency suppressed anti-apoptotic phenotype both in vivo and in vitro (Figs. 4, 5). These data imply that an anti-apoptotic program in the donor’s liver is essential for hepatocyte resistance against IR stress. Similarly, Megan et al. reported that the inactivation of Mcl-1/Bcl-xL in keratinocytes during viral infection promoted GSDME-dependent pyroptosis to release IL1α [27]. Thus, our results are consistent with SIRT1 – Bcl-2/XIAP cross-talk safeguarding against cold stress-mediated activation and IR-triggered sterile inflammation response in the liver.

Enhanced acetylation of the p53 transcription factor in SIRT1-deficient hepatocyte cultures (Supplementary Fig. 3) indicates that SIRT1 can regulate Bcl-2/XIAP anti-apoptotic axis via a deacetylation function in response to cold stress. However, simultaneous senescence signaling may affect SIRT1 regulation of apoptosis because the SIRT1-p53 axis can mediate both cellular senescence and apoptosis pathways, thereby maintaining the genome integrity [33]. Future studies should address the complex interplay between these molecular events.

Consistent with our murine data, hSIRT1 levels associated positively with Bcl-2 expression in human OLTs (Fig. 1) while preventing GSDME processing in cold-stored discarded human livers, confirmed the clinical relevance of SIRT1-mediated multi-faceted cytoprotection. The investigation into the extracellular GSDME-N – IL18 feedback loop revealed that apoptosis and GSDME-mediated PCD became operational during cold activation, both in vivo and in vitro. Recently, machine perfusion has been shown to improve the quality of marginal human liver grafts and extend the preservation time, enabling therapeutic modulation/perfusate biomarker monitoring [34]. Hence, the SIRT1 – Bcl-2/XIAP axis might provide new targets for decreasing the hepatocyte damage in liver transplant patients while disrupting the GSDME-N – IL18 activation axis may promote “rejuvenation” of marginal livers during perfusion preservation.

In summary, hepatocyte SIRT1 – Bcl-2/XIAP crosstalk negatively regulated the apoptosis feedback loop, bridged by GSDME-licensed extracellular IL18 release in IR-stressed OLTs (Fig. 8E). This pathway highlights a novel mechanism in hepatocyte PCD. It provides new putative therapeutic targets against acute or chronic inflammatory liver diseases.

## Materials/subjects and methods

### Clinical liver transplant study

Human studies were approved by the UCLA Institutional Research Board (IRB 13-000143), and written informed consent was received from participants. We performed a retrospective analysis of sixty adult patients who underwent OLT (May 2013-August 2015) and received routine standard of care and immunosuppressive therapy. Recipients who underwent re-transplantation were excluded from the study. Donor livers, procured from donation after brain or cardiac death, were perfused/stored in the UW solution (Niaspan; Bristol-Meyers Squibb). Protocol Tru-Cut needle Bx were obtained from the left lobe after cold storage (at the back table) about 2 h after portal reperfusion (before abdominal closure) and snap-frozen. Hepatic Bx were screened by Western blots (WB) with β-actin normalization for SIRT1, Bcl-2, and cCasp3 expression. Recipient blood samples were evaluated for sALT levels.

### Animals

C57BL/6 male, wild-type (WT; Jackson Laboratory, Bar Harbor, ME), FLOX, and hepatocyte SIRT1-deficient (hSIRT1-KO; NIEHS, Research Triangle Park, NC) mice were used. Animals were housed under pathogen-free conditions, and received care outlined in the Guide for Care and Use of Laboratory Animals (National Academies Press, 2011).

### Mouse model of orthotopic liver transplantation

All mouse experiments were approved by the UCLA Animal Research Committee (ARC 1999-094). We used our mouse model of ex vivo hepatic cold storage and OLT [8]. To mimic marginal human transplantation setting and focus on hepatic SIRT1 while avoiding host alloimmune responses, donor livers (FLOX or hSIRT1-KO; BL6) stored in UW solution (18 h/4 °C) were transplanted to syngeneic recipients. In some experiments, prospective WT recipients of hSIRT1KO liver grafts were conditioned with a neutralizing anti-IL18 Ab (0.5 mg i.p. at 24 h before surgery; YIGI74-1G7; Bio-x-Cell). Liver graft/serum samples were collected at 6 h post-reperfusion. Mice were randomly allocated to the experiment group. No blinding was conducted. Animals with unexpected complications were excluded. To investigate the influence of cold ischemia alone, hepatic tissue/liver flush samples (after infusion of 0.5 ml saline via a portal vein cuff) were collected after cold preservation.

### Hepatocyte isolation and cultures

Primary mouse hepatocytes, isolated by a 2-stage collagenase perfusion method [35], were cultured (3 h/6 h) with/without cold stress (4 °C). Hepatocytes were silenced with siRNA targeting SIRT1, GSDME, Bcl-2, XIAP, and IL18Rβ (Santa Cruz Biochemistry) using Lipofectamine (Invitrogen) before cold stimulation. In some experiments, hepatocytes were pre-treated with a pan-caspase inhibitor (zVAD-FMK; R&D Systems).

### Hepatocyte cell death screening

Cultured hepatocytes were incubated with propidium iodide (PI; 1 μg/ml for 5 min) and screened with a fluorescence microscope for phase-contrast images.

### Statistics

For human data, continuous values were analyzed by the Mann-Whitney *U* test, and categorical variables by Fisher’s exact test. Spearman’s correlation coefficient (r) was used to evaluate the strength of the linear relationship between variables. For mouse data, comparisons between two or multiple groups were assessed using a Student’s t-test and one-way analysis of variance (ANOVA), followed by Tukey’s HSD (honest significance difference) test. All *P* values were 2-tailed and *P* < 0.05 was considered statistically significant. Analyses were performed with GraphPad Prism software. No statistical methods were used to estimate the sample size in advance.

### Supplementary information

Checklist

Supplemental material clean version

Supplemental material original blots file

## Data Availability

All data generated during this study are available from the corresponding author upon reasonable request.
