# Supplementary material for: SIRT1 regulates hepatocyte programmed cell death via GSDME - IL18 axis in human and mouse liver transplantation
Source: Cell Death Dis. 2023 Nov 23;14(11):762. doi: 10.1038/s41419-023-06221-0 (PMC10667508; doi:10.1038/s41419-023-06221-0)
Supplement: Supplementary file 2 — Supplemental material clean version [file 41419_2023_6221_MOESM2_ESM.docx]

**Supplementary Materials**

**SIRT1 Regulates Hepatocyte Programmed Cell Death via**

**GSDME – IL18 Axis in Human and Mouse Liver Transplantation**

Kentaro Kadono^1,2^*, Hidenobu Kojima^1^*, Siyuan Yao^1^, Shoichi Kageyama^1,2^, Kojiro Nakamura^1,2^, Hirofumi Hirao^1^, Takahiro Ito^1^, Kenneth J. Dery^1^, Douglas G. Farmer^1^, Fady M. Kaldas^1^, Xiaoling Li^3^, and Jerzy W. Kupiec-Weglinski^1^

*These authors equally contributed to this work.

^1^ Dumont-UCLA Transplantation Center, Department of Surgery, Division of Liver and Pancreas Transplantation, David Geffen School of Medicine at UCLA, Los Angeles, CA 90095

^2^ Division of Hepato-Biliary-Pancreatic Surgery and Transplantation, Department of Surgery, Graduate School of Medicine, Kyoto University, Kyoto, Japan

^3^ Signal Transduction Laboratory, National Institute of Environmental Health Sciences (NIEHS), Research Triangle Park, NC 27709

**Table of Contents**

**Supplementary Methods……………………………………………………………………2**

**Supplementary Figure 1…………………………………………………………………….4**

**Supplementary Figure 2…………………………………………………………………….5**

**Supplementary Figure 3…………………………………………………………………….6**

**Supplementary Figure 4…………………………………………………………………….7**

**Supplementary Figure 5…………………………………………………………………….8**

**Supplementary Figure 6…………………………………………………………………….9**

**Supplementary Table 1…………………………………………………………………….10**

**Supplementary Table 2…………………………………………………………………….11**

**Supplementary Table 3…………………………………………………………………….12**

**References…………………………………………………………………………………...13**

**Methods**

***Generation of SIRT1 KO mice***

SIRT1 allele with floxed exon4 was backcrossed five times into the C57BL/6 background. It was then bred with mice expressing the Cre recombinase driven by the albumin promoter to generate hepatocyte-specific SIRT1 knockout mice (hSIRT1KO)^1^ or the lysosome promoter for myeloid cell-specific SIRT1 knockout mice (mSIRT1KO)^2^. Their age-matched littermate Lox controls (FLOX) were used as controls.

***Liver warm IRI model***

Mice were anesthetized, injected with heparin (100U/kg), and an atraumatic clip was used to interrupt the hepatic artery/portal venous blood supply to the left/middle liver lobes^3^. After 60min of partial (70%) warm ischemia, the clamp was removed, and mice were sacrificed at 6h of reperfusion.

***Hepatocellular function assay***

Mouse serum AST/ALT levels were measured with Infinity AST/ALT Liquid Stable Reagent (Thermo Fisher Scientific) and validated with Validate GC3 (Maine Standards Company, LLC).

***OLT histology and IRI grading***

Formalin-fixed paraffin-embedded mouse liver sections (5μm) were stained with H&E. The severity of IRI was graded by Suzuki`s criteria^4^.

***Western blot assay***

Proteins were extracted from tissue/cell samples, and their concentration was measured using the BCA Protein Assay kit (Thermo Fisher Scientific). The supernatants were precipitated with ice-cold acetone and resolved in 2×Laemmli buffer. An equal amount of protein was electrophoresed, blotted, incubated with primary Abs, secondary HRP-conjugated Abs, and developed. Each band shows the biological duplicate both *in vivo* and *in vitro*. Primary Abs detecting SIRT1 (9475), caspase-3 (9662), cleaved caspase-3 (9664), caspase-9 (9504), cleaved caspase-8 (9496), HMGB-1 (3935), XIAP (14334), p53 (2524), phosphor-p53 (9284), Acetyl-p53 (2570), Vinculin (18799), β-Actin (12620) (Cell Signaling Technology), caspase-1 (Casper-1) (Adipogen), Bcl-2 (ab182858), GSDME (ab215191), GSDMD (ab209845), IL18 (ab71495), IL18Rβ (ab197832) (Abcam), IL1β (AF-401-NA) (R&D Systems) were used. Original blots are included in another supplementary file.

***Enzyme-linked immunosorbent assay (ELISA)***

Serum concentration of IL18 were measured with ELISA kit (Thermo-Fisher Scientific) according to the manufacture`s protocol.

***qRT-PCR analysis***

RNA extracted with an RNAse Mini Kit (QIAGEN) was reverse transcribed into cDNA. qRT-PCR was performed using QuantStudio 3 (Applied Biosystems). Primers to amplify specific gene fragments are listed (Suppl. Table 3). Target gene expressions were calculated by their ratios to the housekeeping HPRT gene.

***Macrophage cell cultures***

Bone marrow macrophages (BMM) from mouse femurs/tibias were cultured (5×10^6^/well) with 10% L929 medium for 7 days (94-99% CD11b+)^5^ and treated with LPS (100ng/ml; catalog L5418-2ML; Sigma) for 6h.

***Human discarded livers***

We analyzed 6 livers which were procured with the intention of transplantation but declined for clinical use (June, 2018 – May, 2019).

**Supplementary Fig. 1**

**Suppl. Fig. 1. Hepatocyte SIRT1 does not affect canonical inflammasome activation during cold storage of mouse livers:** Groups of FLOX and hSIRT1KO livers cold-stored in UW solution were perfused with physiological saline (0.5ml) via a portal vein-cuff to collect liver flush from supra-hepatic inferior vena cava (n=3/group). WB assisted ditection of Pro-Casp1, Casp1-p20, GSDMD-FL, GSDME-N, Pro-IL1β and IL1β-p17.

**Supplementary Fig. 2**

**Suppl. Fig. 2 SIRT1 promotes anti-apoptotic signaling and prevents GSDME processing in cold-stored discarded human livers: (A)** Discarded human liver grafts (n=6), cold-stored in UW solution for 12h, were divided into two groups based on SIRT1 expression levels. Relative expression levels of Bcl-2, XIAP and GSDME-N/GSDME-FL ratio normalized by β-Actin (*lower left panels*). WB-assited detection of SIRT1, XIAP, Bcl-2, GSDME-FL, GSDME-N and β-Actin (right panels). Data shown are mean±SEM. *p<0.05; **p<0.01 by Student`s t-test. **(B)** Profiles of discarded livers.

**Supplementary Fig.3**

**Suppl. Fig. 3 Ablation of hepatocyte SIRT1 promotes acetylation and phosphorylation of p53 under cold-stress:** Primary mouse hepatocyte cultures conditioned with SIRT1 vs. control siRNA were subjected to cold stimulation. (A) WB assisted-detection of Ac-p53, p-p53 and p53. (B) Relative intensity ratios of Ac-p53/p53 and p-p53/p53. Green line: siControl; purple line: siSIRT1. Data shown are mean±SEM. *p<0.05 by Student`s t-test.

**Supplementary Fig. 4**

**Suppl. Fig. 4. GSDME regulates IL18 release and extrinsic apoptosis signaling in cold-stressed hepatocytes:** Primary mouse hepatocytes pretreated with control or GSDME siRNAs were subjected to 6h cold stress in vitro. **(A)** WB-assisted detection of GSDME-FL, GSDME-N, Pro-IL18, IL18, HMGB1, Bid, t-Bid, Pro-Casp3, cCasp3, PARP, cPARP and β-Actin in cell lysates (*left panel*) and IL18 and HMGB1 in supernatants (*right panel*). **(B)** Relative intensity of IL18 and HMGB1 in supernatants. **(C)** Relative intensity of GSDME-FL, t-Bid/Bid and cCasp3/Pro-Casp3 normalized by β-Actin. Data shown are mean±SEM. *p<0.05; **p<0.01 by Student`s t-test.

**Supplementary Fig. 5**

**Suppl. Fig. 5. Hepatocyte SIRT1 fails to regulate canonical inflammasome activation in mouse OLT: (A-B)** FLOX and hSIRT1 liver grafts, stored in UW solution (4℃/18h), were transplanted into WT mice, followed by OLT sampling at 6h post-reperfusion. **(A)** WB detection of Pro-Casp1, Casp1-p20, GSDMD-FL and GSDMD-N. **(B)** Relative intensity of Casp1-p20/Pro-Casp1 ratio in OLT. **(C-D)** Primary mouse hepatocytes conditioned with SIRT1 or control siRNA were subjected to cold stimulation for the indicated time periods. **(C)** WB assisted detection of Pro-Casp1 and Casp1-p20. **(D)** Relative intensity ratios of Casp1-p20/Pro-Casp1.

**Supplementary Fig. 6**

**Suppl. Fig. 6. Myeloid SIRT1 fails to regulate apoptosis in IR-stressed mouse livers:** FLOX and myeloid-specific SIRT1 KO (mSIRT1) mice were subjected to 60min warm ischemia followed by 6h reperfusion. **(A)** WB-assisted detection of SIRT1, cCasp3 and Vinculin as a loading control. **(B)** Relative intensity in SIRT1 and cCasp3 normalized by Vinculin.

| **Supplementary Table 1**  **Human liver transplant recipients` perioperative variables (60 clinical cases)** | | | |
| --- | --- | --- | --- |
| **Variables** | **SIRT1 low (n=30)** | **SIRT1 high (n=30)** | **p value** |
| Age (years) | 60 (23-73) | 57 (29-73) | 0.5248 |
| Sex (M/F) | 17 (56.7%)/13 (43.3%) | 23 (76.7%)/7 (23.3%) |  |
| Race |  |  |  |
| White | 18 | 16 |  |
| Hispanic | 10 | 11 |  |
| Black | 0 | 0 |  |
| Asian | 1 | 1 |  |
| Others | 1 | 2 |  |
| Height (cm) | 167.6 (149.9-191.0) | 175.3 (149.9-193.0) | 0.512 |
| BMI (kg/m^2^) | 28.48 (20.66-44.30) | 24.65 (14.51-47.54) | 0.1731 |
| Disease etiology |  |  |  |
| HBV | 3 | 2 |  |
| HCV | 12 | 12 |  |
| EtOH | 3 | 6 |  |
| Cryptogenic cirrhosis/NASH | 1 | 1 |  |
| ALF | 1 | 0 |  |
| Others | 10 | 9 |  |
| HCC (with/without) | 12 (40%)/18 (60%) | 12 (40%)/18 (60%) |  |
| ABO |  |  |  |
| Identical | 27 (90%) | 29 (96.7%) |  |
| Compatible | 3 (10%) | 1 (3.3%) |  |
| MELD score | 31 (9-44) | 31 (14-42) | 0.528 |
| Pretransplant AST (IU/L) | 66.5 (22-6967) | 61.00 (23.00-1231) | 0.5252 |
| Pretransplant ALT (IU/L) | 43.5 (9-6168) | 34.5 (11-617) | 0.3756 |
| Pretransplant T-Bil (mg/dL) | 7.1 (0.7-40.70) | 5.6 (0.3-59.10) | 0.8978 |
| Preoperative Alb | 3.45 (2.6-6.8) | 3.7 (1.6-6.1) | 0.8464 |
|  |  |  |  |
|  |  |  |  |
|  |  |  |  |
|  |  |  |  |

| **Supplementary Table 2**  **Donors` perioperative variables (60 clinical cases)** | | | |
| --- | --- | --- | --- |
| **Variables** | **SIRT1 low (n=30)** | **SIRT1 high (n=30)** | **p value** |
| Age (years) | 43 (13-72) | 42 (15-74) | 0.98 |
| SEX (M/F) | 16 (53.3%)/14 (46.7%) | 13 (43.3%)/17 (56.7%) |  |
| BMI (Kg/m^2^) | 25.63 (13.4-42.58) | 26.58 (19.64-32.59) | 0.1731 |
| Pre-transplantation AST | 39.50 (10.0-306.0) | 35.0 (12.0-314.0) | 0.8978 |
| Pre-transplantation ALT | 28.0 (8.0-403.0) | 23.5 (8.0-730.0) | 0.6568 |
| Cold Ischemia Time (min) | 480 (150-1213) | 435 (120-750) | 0.3013 |
| Warm Ischemia Time (min) | 50.5 (23-79) | 54 (25-78) | 0.5128 |
|  |  |  |  |
|  |  |  |  |

| **Supplementary Table 3**  **Primer Sequences Used for Real-Time Quantitative PCR (mouse study)** | | |
| --- | --- | --- |
| **Gene** | **Forward** | **Reverse** |
| IL10 | 5`-CGACTCCTTAATGCAGGACT-3` | 5`-TTGATTTCTGGGCCATGC-3` |
| IL13 | 5`-CCTGGCTCTTGCTTGCCTT-3` | 5`-GGTCTTGTGTGATGTTGCTCA-3` |
| IL4 | 5`-GGTCTCAACCCCCAGCTAGT-3` | 5`-GCCGATGATCTCTCTCAAGTGAT-3` |
| CXCL10 | 5`-GCTGCCGTCATTTTCTGC-3` | 5`-TCTCACTGGCCCGTCATC-3` |
| TNFα | 5`-CCTATGTCTCAGCCTCTCT-3` | 5`-TTGGGAACTTCTCATCCCTT-3` |
| Bcl-2 | 5`-CCTGTGGATGACTGAGTACCTG-3` | 5`-AGCCAGGAGAAATCAAACAGAGG-3` |
| Mcl-1 | 5`-AGCTTCATCGAACCATTAGCAGAA-3` | 5`-CCTTCTAGGTCCTGTACGTGGA-3` |
| XIAP | 5`-GGCAGAATATGAAGCACGGATCG-3` | 5`-CACTTGGCTTCCAATCCGTGAG-3` |
| NLRP3 | 5`-TCACAACTCGCCCAAGGAGGAA-3` | 5`-AAGAGACCACGGCAGAAGCTAG-3` |
| IL1β | 5`-TGTAATGAAAGACGGCACACC-3` | 5`-TCTTCTTTGGGTATTGCTTGG-3` |
| IL18 | 5`-GACAGCCTGTGTTCGAGGATATG-3` | 5`-TCTTCTTACAGGAGAGGGTAGAC-3` |
| IL18Rβ | 5`-ACAACACGGACCATACGGCTGA-3` | 5`-GTACCAGTAGAGGAAAGCAGCTG-3` |
|  |  |  |
|  |  |  |
|  |  |  |
|  |  |  |
|  |  |  |
|  |  |  |
|  |  |  |
|  |  |  |
|  |  |  |
|  |  |  |
|  |  |  |
|  |  |  |
|  |  |  |
|  |  |  |

**REFERENCES**

**1.** Purushotham A, Schug TT, Xu Q, Surapureddi S, Guo X, Li X. Hepatocyte-specific deletion of SIRT1 alters fatty acid metabolism and results in hepatic steatosis and inflammation. Cell Metab 2009;9:327-338.

**2.** Schug TT, Xu Q, Gao H, Peres-da-Silva A, Draper DW, Fessler MB*, et al.* Myeloid deletion of SIRT1 induces inflammatory signaling in response to environmental stress. Mol Cell Biol 2010;30:4712-4721.

**3.** Shen XD, Ke B, Zhai Y, Gao F, Anselmo D, Lassman CR*, et al.* Stat4 and Stat6 signaling in hepatic ischemia/reperfusion injury in mice: HO-1 dependence of Stat4 disruption-mediated cytoprotection. Hepatology 2003;37:296-303.

**4.** Shohachi S, Luis H T-P, Francisco J R, Dolores C. Neutrophil infiltration as an important factor in liver ischemia and reperfusion injury. Modulating effects of FK506 and cyclosporine. Transplantation 1993;55:1265–1272.

**5.** Nakamura K, Zhang M, Kageyama S, Ke B, Fujii T, Sosa RA*, et al.* Macrophage heme oxygenase-1-SIRT1-p53 axis regulates sterile inflammation in liver ischemia-reperfusion injury. J Hepatol 2017;67:1232-1242.
