# Supplementary material for: SIRT1 regulates hepatocyte programmed cell death via GSDME - IL18 axis in human and mouse liver transplantation
Source: Cell Death Dis. 2023 Nov 23;14(11):762. doi: 10.1038/s41419-023-06221-0 (PMC10667508; doi:10.1038/s41419-023-06221-0)

Fig.2 Original Blots

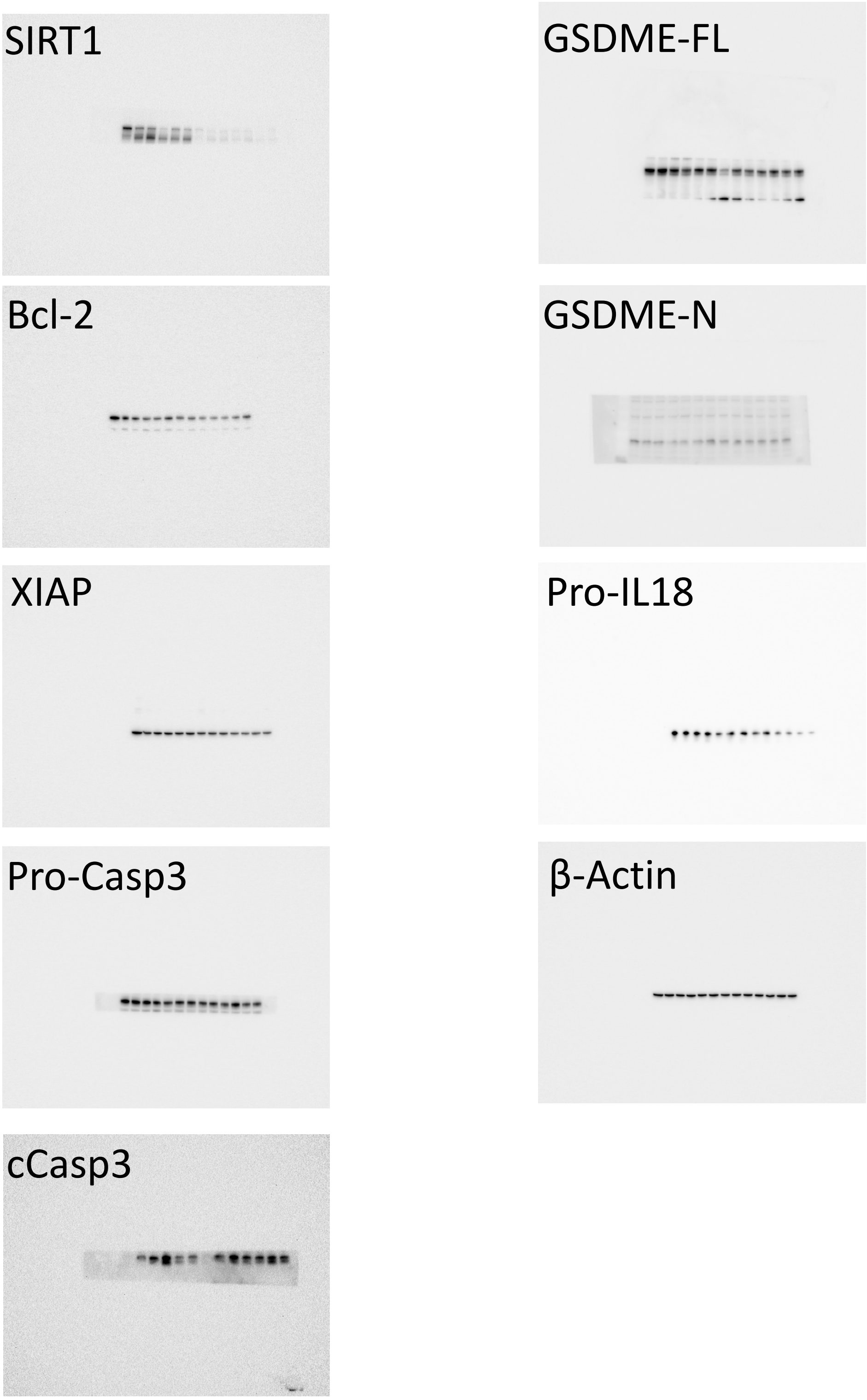

Fig.3 Original Blots

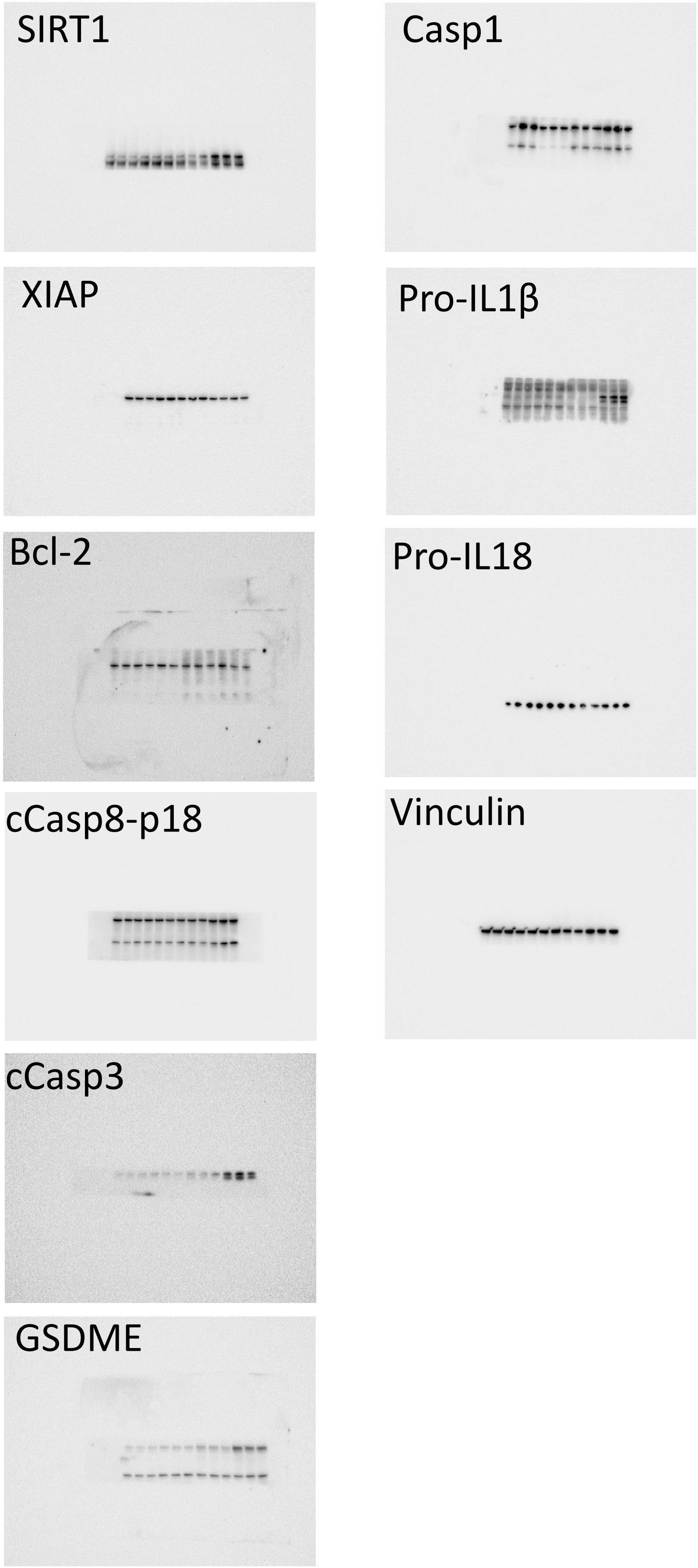

Fig.4 Original Blots

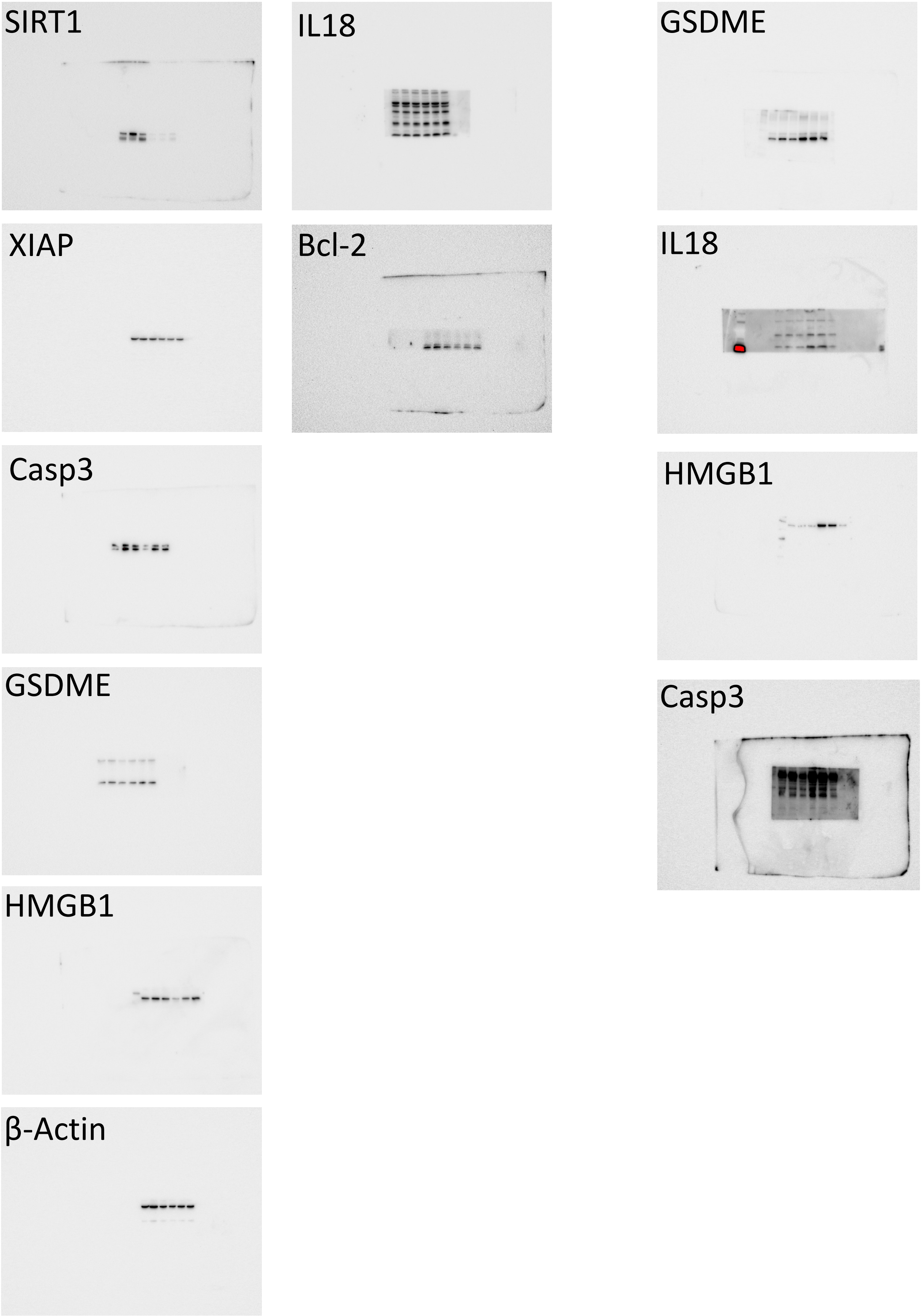

Fig.5 Original Blots

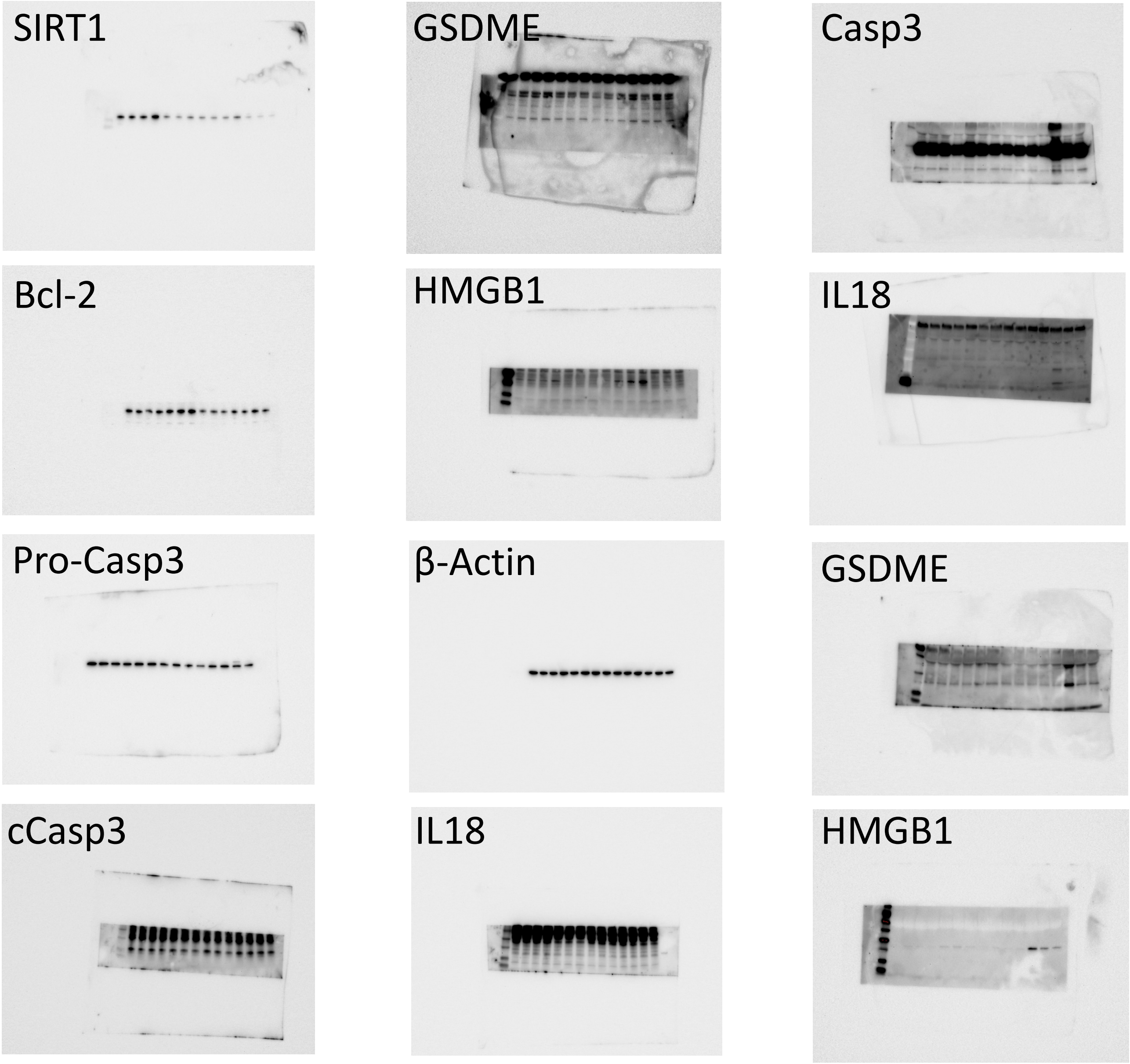

Fig.6 Original Blots

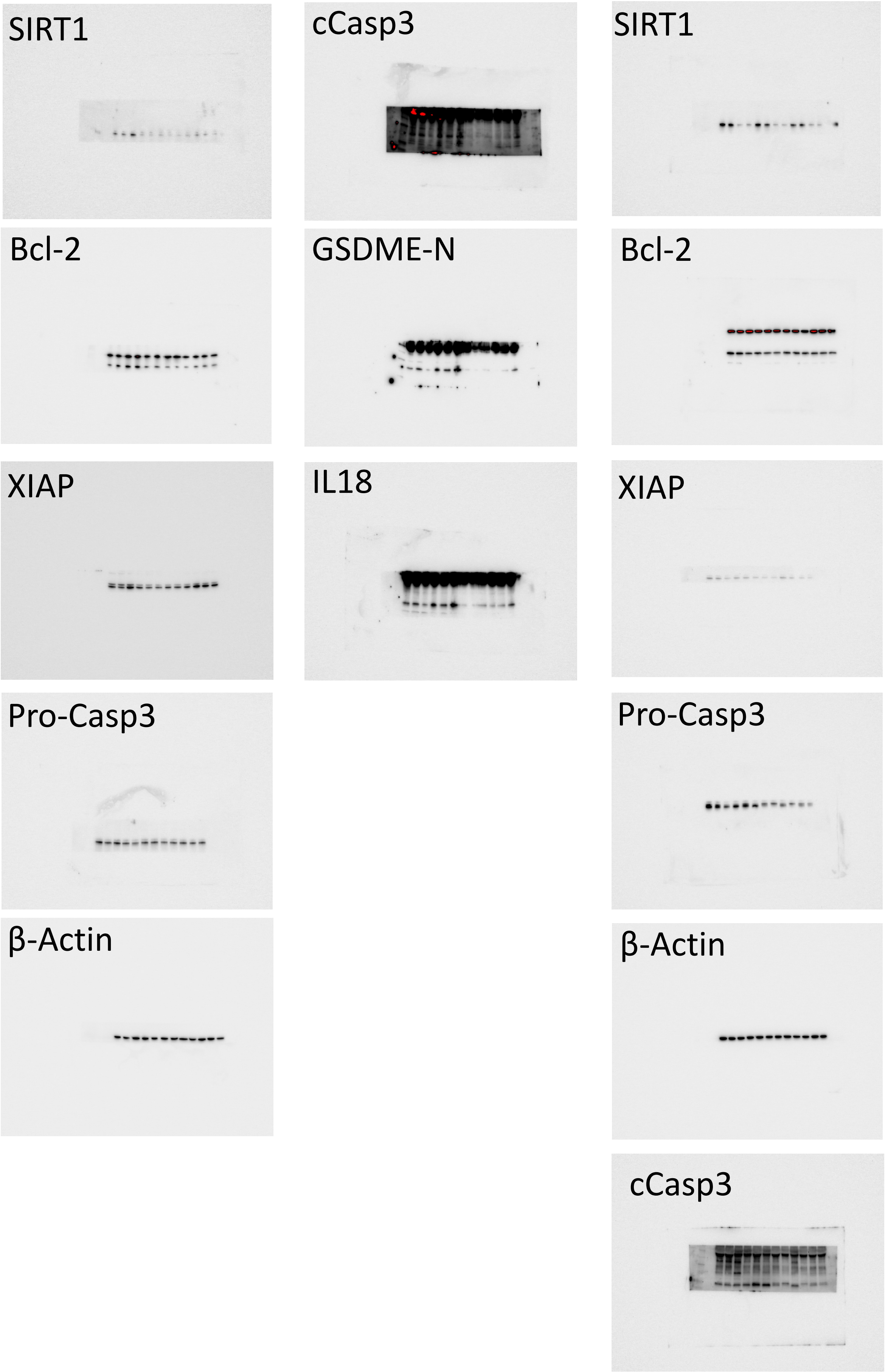

Fig.7 Original Blots

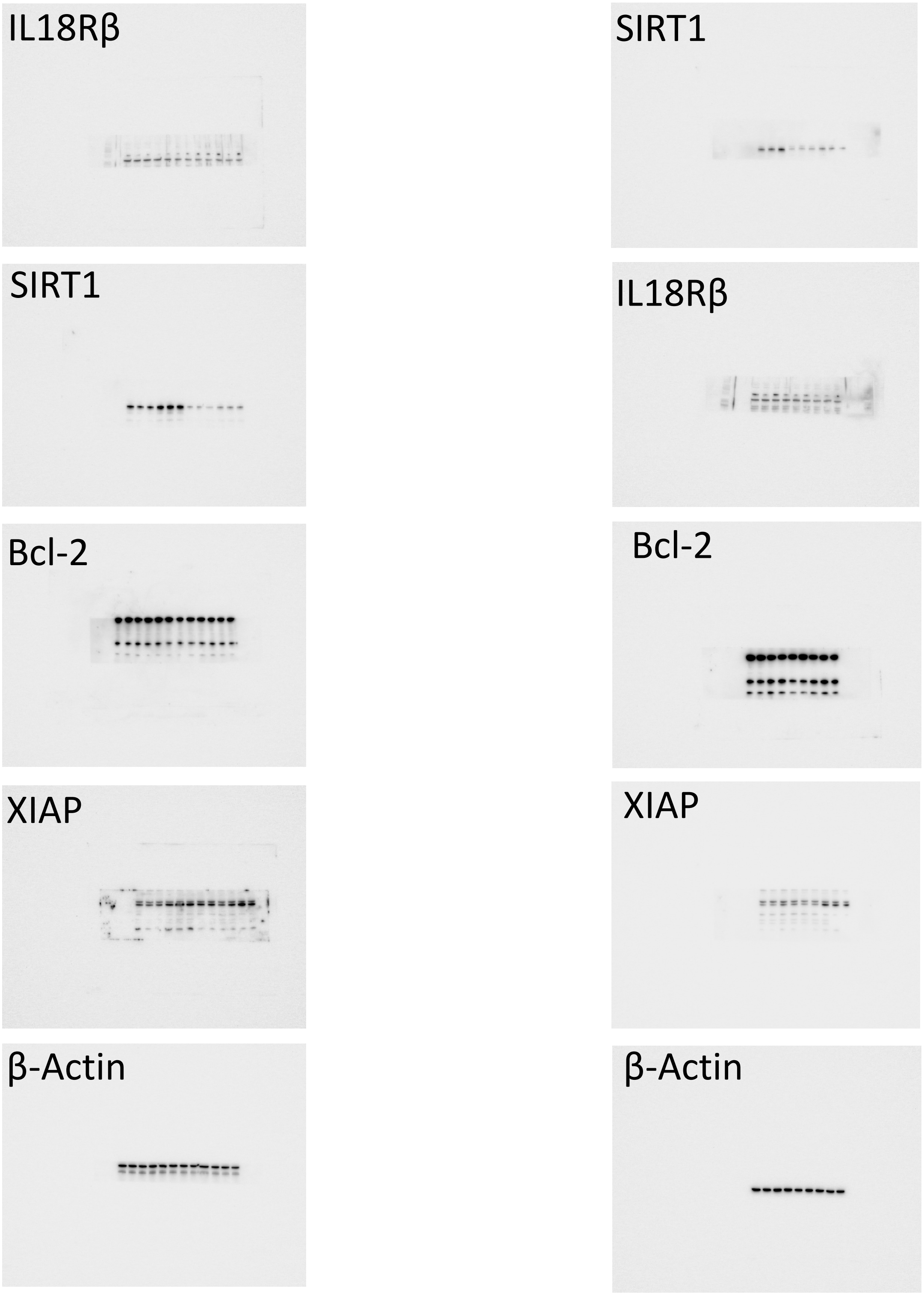

Fig.8 Original Blots

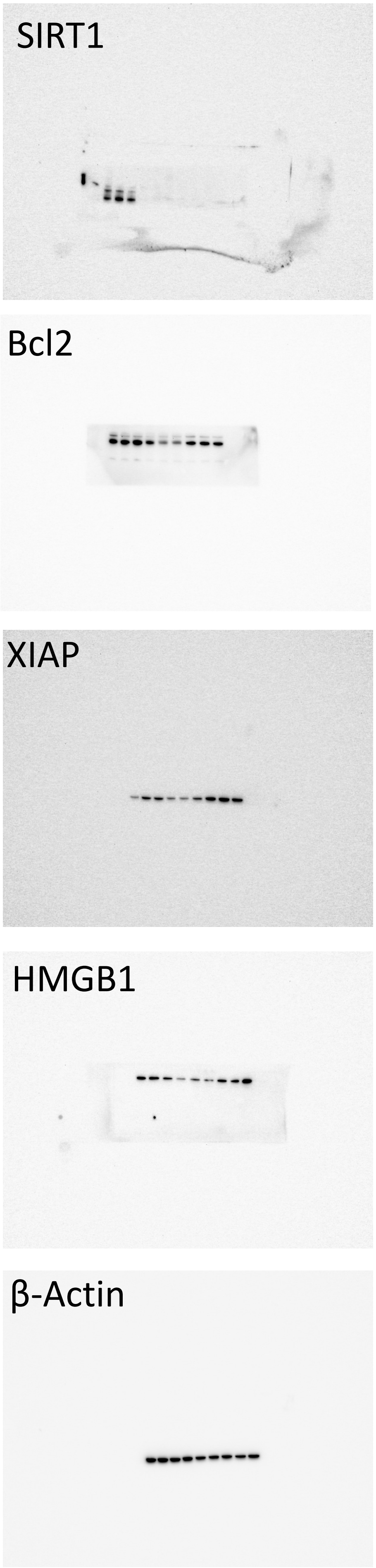

Supplement: Supplementary file 3 — Supplemental material original blots file [file 41419_2023_6221_MOESM3_ESM.pdf]
